# Associations of ANGPT2 expression and its variants (rs1868554 and rs7825407) with multiple myeloma risk and outcome

**DOI:** 10.3389/fonc.2025.1468373

**Published:** 2025-03-06

**Authors:** Sylwia Popek-Marciniec, Wojciech Styk, Sylwia Chocholska, Aneta Szudy-Szczyrek, Katarzyna Sidor, Grazyna Swiderska-Kolacz, Marek Hus, Joanna Czerwik-Marcinkowska, Szymon Zmorzynski

**Affiliations:** ^1^ Laboratory of Genetics, Academy of Zamosc, Zamosc, Poland; ^2^ Academic Laboratory of Psychological Tests, Medical University, Lublin, Poland; ^3^ Chair and Department of Hematooncology and Bone Marrow Transplantation, Medical University of Lublin, Lublin, Poland; ^4^ Institute of Biology, Jan Kochanowski University, Kielce, Poland

**Keywords:** hematology malignancies, plasma cell, angiogenesis, polymorphisms, thalidomide, bortezomib

## Abstract

The growth of blood vessels from the existing vasculature has a significant impact on the course of multiple myeloma (MM). The ANGPT2 (angiopoietin-2) protein is encoded by the *ANGPT2* gene and plays an important role in angiogenesis. The expression of proangiogenic proteins is influenced not only by microenvironmental factors but also by genetic changes. We analyzed two variants/polymorphisms of the *ANGPT2* gene, rs1868554 (T>A) and rs7825407 (G>C). Both are located in the intron sequence and can affect the final mRNA sequence by modifying splicing.

**Purpose:**

Therefore, we assessed the impact of selected variants on *ANGPT2* gene expression at the mRNA and protein levels. Additionally, we evaluated the associations of the analyzed genetic changes with the clinical and laboratory parameters of the disease and the response to bortezomib/thalidomide-based therapies. We hypothesize that variants and expression of the *ANGPT2* gene may be associated with a greater risk of MM development and may also affect the response to treatment in MM patients.

**Patients and methods:**

Genomic DNA extracted from 103 newly diagnosed MM patients and 120 healthy blood donors was used to analyze *ANGPT2* variants (via automated DNA sequencing). RNA was subjected to real-time PCR to determine *ANGPT2* expression at the mRNA level. The concentration of angiopoietin-2 (in MM sera) was determined by ELISA.

**Results:**

The results of our study showed that individuals with the AA genotype of rs1868554 and the CC genotype of rs7825407 had a greater risk of developing MM (OR=6.12, p=0.02 and OR=6.01, p=0.02, respectively). The *ANGPT2* gene variants did not affect ANGPT2 expression at the mRNA level. However, *ANGPT2* expression was positively correlated with CRP (Spearman’s rho 0.26, p<0.05) and negatively correlated with LDH (Spearman’s rho -0.25, p<0.05) in MM patients.

**Conclusion:**

Our results showed that *ANGPT2* expression at the mRNA level correlates with CRP, a negative prognostic factor in MM. The ANGPT2 protein is a proangiogenic factor, and its concentration is significantly greater in MM patients than in healthy individuals, which was also confirmed in our research. Therefore, this protein with VEGF and HB-EGF, should be considered in the future as a markers of angiogenesis in MM.

## 1 Introduction

Angiogenesis is the most important condition for malignant cell development and proliferation. Tumors larger than 1-2 mm will not be able to continue growing if blood vessels do not supply them with nutrients (1, 2). This indicates that the process of tumor angiogenesis is essential for tumor growth, expansion and overall progression. The above changes are also specific to multiple myeloma (MM). The development of blood vessels from the existing vasculature also contributes to the progression of this disease (2). MM is a hematologic malignancy characterized by clonal expansion of plasma cells in the bone marrow (BM), osteolytic bone disease and monoclonal gammopathy (3). In the course of MM, angiogenesis can be assessed by measuring microvascular density (MVD). The mean number of immunohistochemically stained microvessels in paraffin-embedded BM sections was counted (4, 5). The bone marrow microenvironment plays an important role in angiogenesis, MM progression and response to therapeutic agents (6). One of the factors involved in the process of BM angiogenesis is angiopoietin-2 (ANGPT2, Ang-2) (7, 8). It is a protein that regulates vessel growth and maturation during angiogenesis. ANGPT2 binds to the TIE2 receptor and cooperates with the VEGF pathway to maintain physiological functions. Furthermore, in previous studies, a significant increase in circulating angiopoietin-2 in MM patients compared to healthy individuals was observed (4, 9). A hypoxic microenvironment is a common and important feature of most malignancies. Hypoxia affects the cell phenotype and mediates the response to the effects of chemotherapy, radiotherapy and immunotherapy on tumor cells (10). Under hypoxic conditions, HIF-1 induces increased expression of the *ANGPT2* gene (8). In addition, to date, ANGPT2 expression has been demonstrated to be associated with disease severity (4, 9). ANGPT2 produced in the BM microenvironment may contribute to the development of MM angiogenesis, and this molecule may be further useful as both a biomarker of angiogenesis and a potential therapeutic target (11). The involvement of MM cells in ANGPT2 synthesis is still controversial. Hoffamn et al. showed that ANGPT2 has prognostic significance. Three angiogenesis markers, EGF, HGF and ANGPT2, are associated with progression from monoclonal gammopathy of undetermined significance (MGUS) to MM (12).

The *ANGPT2* gene is located on chromosome 8 (*locus* 8p23.1) and is known to be mostly expressed by activated endothelial cells under physiological conditions (13). Lohr et al. observed homozygous deletions of the 8p23.1 *locus* in MM patients (14). Considering these findings, we analyzed the *ANGPT2* gene located in this region, whose product may be related to angiogenesis. The ANGPT2 protein is a key mediator of angiogenesis. Studies on lung adenocarcinoma have shown that the ANGPT2 protein level is directly proportional to the size of the tumor and is correlated with the presence of metastases. More importantly, a higher concentration of angiopoietin-2 was negatively correlated with the overall survival (OS) of individuals with lung adenocarinoma (8). Increased ANGPT2 expression has also been described as a potential prognostic factor in colorectal carcinoma (13), gastric cancer (15), hepatocellular cancer (16, 17), lung cancer (18), and chronic lymphocytic leukemia (19). In MM, the serum ANGPT2 level is correlated with disease progression and response to therapy (9, 11).

Very little is known about the effects of single-nucleotide polymorphisms/variants (SNPs) in angiogenesis-related genes on treatment outcomes in MM patients. Genetic variants in the *ANGPT2* gene may lead to its altered expression and can affect protein activity. For our research, we selected two variants, rs1868554 (T>A) and rs7825407 (G>C), which are located in the intron of the *ANGPT2* gene. Variants that are present in introns do not necessarily directly affect the amount of transcript but can affect the final mRNA sequence by modifying splicing (20). The selected variants have not been studied in MM. Meyer and colleagues demonstrated that rs1868554 alters the ratio of ANGPT2 isoforms (20). Several studies have shown that *ANGPT2* gene variants are associated with the course and effects of treatment for colorectal cancer (21), lung cancer (22), head and neck squamous cell carcinoma (23), hepatocellular carcinoma (24) and diseases other than cancer, such as rheumatoid arthritis (25) and systemic sclerosis (26).

Therefore, we wanted to determine whether the selected variants of the *ANGPT2* gene have an impact on ANGPT2 expression. Moreover, we hypothesize that the rs1868554 and rs7825407 variants may be associated with a greater risk of developing MM and may also affect the response of MM patients to treatment. The aim of our research was also to determine whether *ANGPT2* variants have a significant impact on the course of the disease, including clinical and laboratory MM parameters, as well as the response to bortezomib/thalidomide-based therapies.

## 2 Materials and methods

### 2.1 Patients and samples

The study enrolled 223 unrelated individuals, including 103 newly diagnosed MM patients and 120 healthy blood donors. The study participants were selected from the same ethnicity (Caucasian population). Between 2013 and 2020, material (bone marrow aspirates, peripheral blood and plasma) from MM patients was obtained from the Chair and Department of Hemato-Oncology and Bone Marrow Transplantation (Medical University of Lublin, Poland) by clinicians. The characteristics of the MM patients are presented in **Table 1**. MM bone marrow aspirates were used for cytogenetic analysis and *ANGPT2* gene expression determination at the mRNA level; sera were used for ANGPT2 protein concentration analysis; and peripheral blood was used for *ANGPT2* gene genotyping.

**Table 1:** The characteristics of the MM patients included in the study.

Variables	MM patients, n=103
Age (years)	65.02
Sex	
Male	52
Female	51
Type of MM*	
IgG	62
IgA	21
Light chain	20
Stage according to the International Staging System*	
I	29
II	27
III	47
Smoking	
Yes	19
No: Nonsmokers	74
No: Ex-smokers	10
Exposure to carcinogenic factors	
Yes	25
No	78
Additionally, other type of cancer	
Yes	7
No	96
Renal failure*	
No	83
Yes	20
The stage of chronic kidney disease (grade)	
G1	35
G2	27
G3A	14
G3B	10
G4	7
G5	10
Anemia grade before treatment (WHO)	
Absent	29
I – mild	37
II – moderate	25
III – severe	12
Structural cytogenetic changes*	
del(17p13.1)	11
del(17p13.1) and t(4;14)	6
del(17p13.1) and t(14;16)	1
t(4;14)	10
t(14;16)	1
Chromosome 17 aneuloidies	
Present	16
Absent	87
*NOTCH1* mutational status (by automated DNA sequencing) of exon 26 (457 bp), exon 27 (351) and exon 34 (PEST domain – 489 bp).	
No	103
Yes	0
Chemotherapy	
Cyclophosphamide, Thalidomide, and Dexamethasone (CTD)	47
Velcade, Cyclophosphamide, Dexamethasone (VCD), and VD	31
Velcade, Thalidomide, and Dexamethasone (VTD)	23
Died before chemotherapy	2

*at diagnosis.

The control group (for genotyping analysis) consisted of healthy blood donors (60 men and 60 women, with a mean age of 37.9 years) from the Regional Blood Donation and Blood Treatment Center in Kielce, Poland.

**Figure 1:**
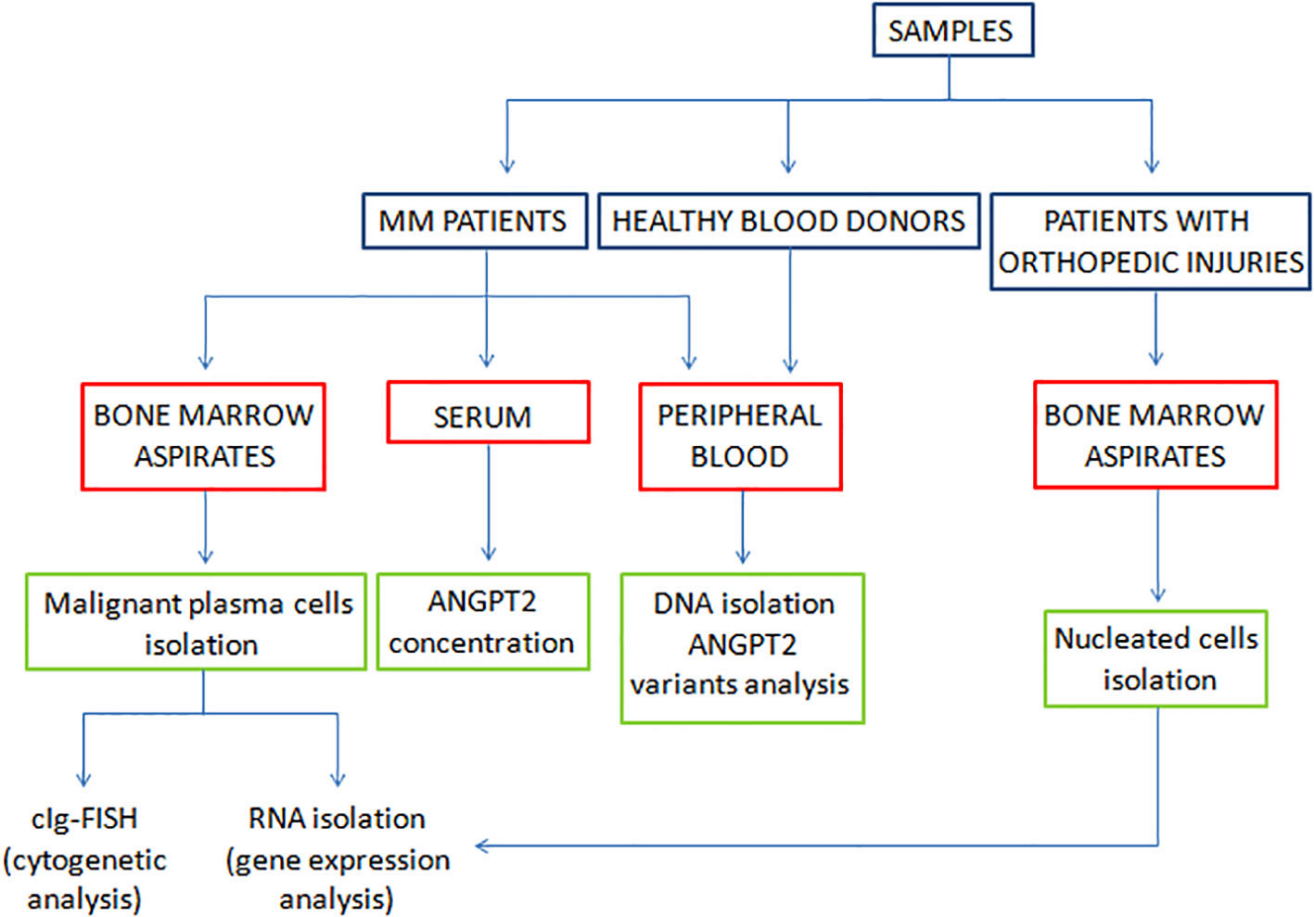
Experiment overflow.

Bone marrow aspirates obtained from 18 non-neoplastic patients with orthopedic injuries who were hospitalized at the Department of Orthopedic and Trauma Surgery of Jan Kochanowski University of Kielce were used for the expression study. Nucleated cells were isolated from bone marrow aspirates for gene expression analysis. In this regard, plasma cells were isolated from MM bone marrow aspirates via a magnetic method with CD138+ beads according to the manufacturer’s protocol (Miltenyi Biotec, Bergisch Gladbach, Germany). From bone marrow aspirates obtained from nonneoplastic patients with orthopedic injuries, mononuclear cells were isolated with Lymphoprep (Serumwerk, Bernburg, Germany) and used to determine *ANGPT2* gene expression at the mRNA level. The experimental overflow is shown in **Figure 1**.

The study obtained positive opinions from the Bioethics Committee at the Medical University of Lublin (No. KE-0254/165/2013, No. KE-0254/337/2016) and the Bioethics Committee at Jan Kochanowski University of Kielce (No. KB-41/2016), according to the ethical standards established by the Helsinki Declaration. All methods were performed in accordance with the relevant guidelines and regulations. All study participants provided written informed consent. The inclusion and exclusion criteria for all individuals in the study are described in **Table 2**.

**Table 2:** Inclusion and exclusion criteria for MM patients and healthy blood donors.

Inclusion Criteria for MM Patients and Healthy Blood Donors	Exclusion Criteria for MM Patients
‐ Signed informed consent. ‐ 18 years of age or older. ‐ Successful genotyping.	‐ Active smoldering MM. ‐ Active plasma cell leukemia. ‐ Documented systemic amyloid light chain amyloidosis. ‐ Active central nervous system involvement with MM.
Additional inclusion criteria for MM patients	Exclusion criteria for healthy donors
‐ Newly diagnosed disease. ‐ Measurable disease for secretory, poor secretory and nonsecretory MM. For secretory MM, the presence of quantifiable monoclonal component, ≥0.5 g/dL, was taken into account. For poor secretory or nonsecretory MM, the level of the affected serum free light chain must be ≥10 mg/dL or ≥100 mg/L with an abnormal free light-chain ratio. ‐ Eastern Cooperative Oncology Group (ECOG) Performance status ≤3. ‐ Life expectancy more than 3 months.	‐ HIV infection, hepatitis B or C infection. ‐ Bacterial infection resulting in the development of syphilis or tuberculosis. ‐ Severe coagulation disorders (e.g., hemophilia) or significantly impaired venous access. ‐ A condition that requires active medical intervention or monitoring to avert serious danger to the participant’s health or well-being.

### 2.2 DNA isolation

DNA was isolated from the peripheral blood of healthy blood donors and MM patients. For this purpose, a commercial kit (Qiagen, Velno, Netherlands) was used according to the manufacturer’s recommendations. The concentration and quality of the obtained nucleic acid were then checked using a NanoDrop One device (Thermo Fisher Scientific, Waltham, MA, USA).

### 2.3 *ANGPT2* genotyping

**Figure 2:**
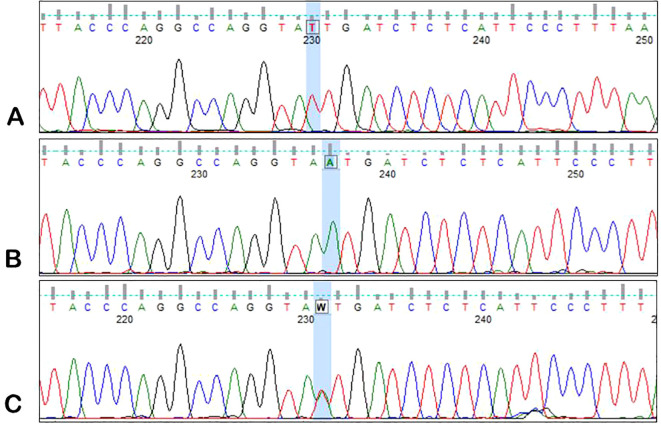
Sample electrophoregrams showing individual variants of the rs1868554 polymorphism of the *AGPT2* gene. **(A)** – T/T variant, **(B)** – A/A variant, **(C)** – T/A variant.

**Figure 3:**
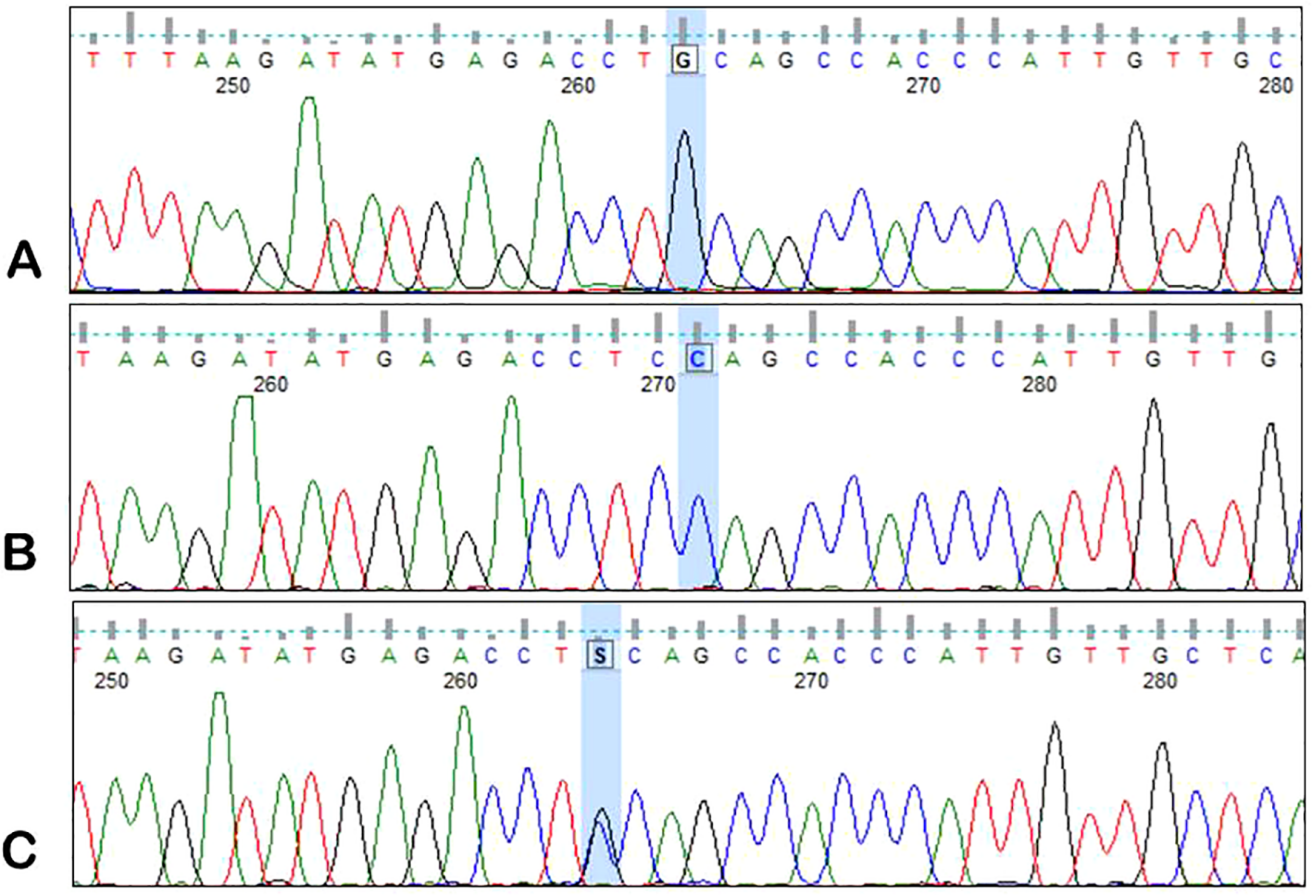
Sample electrophoregrams showing individual variants of the rs7825407 polymorphism of the *AGPT2* gene. **(A)** – G/G variant, **(B)** – C/C variant, **(C)** – G/C variant.

DNA obtained from peripheral blood samples (from MM patients and healthy blood donors) was used to analyze the *ANGPT2* rs1868554 and rs7825407 variants. *ANGPT2* status was determined by automated DNA sequencing. A fragment of the intron (of the *ANGPT2* gene) was amplified by PCR using a T100 Thermal Cycler™ (Bio-Rad, California, USA) with the following primers: forward, 5′-CAGTTAACTTGGGAGGCTTAGTG-3′; reverse, 5′-TGGCCTACGTCTTCTTGAGTC-3′. Each PCR mixture (10 µl) contained 100 ng of genomic DNA, RUN reaction buffer (A&A Biotechnology), a dNTP mixture (0.25 mM), RUN polymerase (0.25 U) (A&A Biotechnology) and primers (10 µM each). The mixture was heated at 95°C for 5 min, after which 35 amplification cycles were performed: denaturation at 95°C for 20 s, annealing at 60°C for 20 s, and elongation at 72°C for 30 s. The final elongation time was 5 min at 72°C. Sequencing PCR and analysis of the results were performed as previously described by using the BigDye Terminator v3.1 Cycle Sequencing Kit (Applied Biosystems) (27). The results were analyzed with the use of Applied Biosystems software – Data Collection Software version 3.1 (**Figures 2**, **3**).

### 2.4 cIg-FISH method

Chromosomal aberrations with prognostic significance in MM were tested using cytoplasmic immunoglobulin and FISH (cIg-FISH) in accordance with the recommendations of Ross et al., 2012 (28). The culture and staining of malignant plasma cells were performed according to a previously described protocol with modifications (29, 30). The analysis of the results with the probes used was carried out as previously described (31).

### 2.5 RNA isolation and *ANGPT2* expression

RNA isolation from mononuclear bone marrow cells was carried out with a total RNA midi kit (Aabiot, Gdansk, Poland). The concentration of isolated RNA was checked spectrophotometrically using a NanoDrop One device (Thermo Scientific, Waltham, MA, USA). The quality of these nucleic acids was checked via electrophoresis on a 2% agarose gel. The RNA was stored at −80°C.

A reverse transcription reaction (RT−PCR) was performed after RNA isolation. RT−PCR was performed using a T100™ Thermal Cycler (Bio-Rad, California, USA). Real-time PCR was carried out on the cDNA (100 ng) template. Real-time PCR was performed using SYBR Green RT−PCR Mix (Aabiot, Poland), an annealing temperature of 60°C, and 100 μM primers (Genomed, Warszawa, Poland): forward, 5′-CAGTTAACTTGGGAGGCTTAGTG-3′; reverse, 5′-TGGCCTACGTCTTCTTGAGTC-3′. Twenty microliters of the reaction mixture was transferred to each well of the PCR plate. The plate was centrifuged and placed in a CFX Opus 96 Real-Time PCR System (Bio-Rad, California, USA). The qRT−PCR mixture was prepared according to the manufacturer’s protocol (A&A Biotechnology, Gdansk, Poland). Every sample was assayed in duplicate, and expression was calculated according to the 2^−ΔΔCt^ method—normalized expression (32). The expression values are presented as the logarithm of R to base 2, where R was calculated as follows: R = 2^−ΔΔCt^, ΔΔCt = ΔCt of the control −ΔCt of the analyzed gene, and every ΔCt = Ct of the analyzed gene − Ct of the endogenous control. The expression of GAPDH served as a control. For this purpose, 100 μM primers (Genomed, Warszawa, Poland) were used with an annealing temperature of 60°C: forward, 5′-CAACGGATTTGGTCGTATTG-3′; reverse, 5′-GGATCTCGCTCCTGGAAG-3′.

A normalized expression (R) in the range of 0.8–1.2 indicated a normal level of gene expression, R < 0.8 indicated low expression, and R > 1.2 indicated high expression (33, 34).

### 2.6 Enzyme-linked immunosorbent assay

A specific ELISA kit (Invitrogen, Waltham, MA, USA) was used (according to the manufacturer’s protocol) to determine the level of angiopoietin-2 in serum samples collected from 70 MM patients. A Multiskan FC plate reader (Thermo Scientific, Waltham, MA, USA) at a wavelength of 450 nm was used for the measurement of angiopoietin-2. The serum samples were diluted 20 times. The concentration read from the standard curve was multiplied by the dilution factor (2×).

### 2.7 Statistical analysis

The laboratory results of MM patients were compared with those of patients with the studied *ANGTPT2* gene genotypes using t tests (for continuous variables) and chi-square tests (for categorical variables). The associations between *ANGTPT2* genotypes and clinical data were assessed using the chi-square test or Fisher’s exact test (when one expected value was <5). Quantitative data are presented as frequencies or percentages. Hardy–Weinberg equilibrium (HWE) was assessed using the chi-square test with Yates correction for groups of fewer than five individuals. For the 95% confidence intervals (CIs), we assumed *p* = 0.05 and χ^2^ = 3.84; thus, if χ^2^ ≤ 3.84 and corresponding *p* ≥ 0.05, then the population was in HWE. The Cox proportional hazard model was used for univariate and multivariate analyses of OS and progression-free survival (PFS). The Kaplan–Meier method and log-rank test were used for survival analysis. Pearson correlation analysis was used to evaluate the associations between the *ANGTPT2* expression level and laboratory/clinical data. We assumed a 5% error of inference and an associated significance level of *p* < 0.05, indicating the existence of statistically significant differences. Statistical analyses were performed using Statistica ver. 12.5 (StatSoft, Krakow, Poland).

## 3 Results

The present study included 103 MM patients (52 males and 51 females). The rs1868554 and rs7825407 variants and the expression of the *ANGPT2* gene at the mRNA and protein levels were analyzed.

### 3.1 Frequencies of alleles and genotypes and their association with MM risk

Genotyping was successful for all the individuals investigated within the study. This was one of the inclusion criteria for MM patients and healthy blood donors. In MM patients, the *ANGPT2* variants were not in the Hardy−Weinberg equilibrium (**Table 3**). The population of healthy blood donors was in HWE. In the dominant and recessive models, AA homozygotes of rs1868554 and CC homozygotes of rs7825407 were associated with a greater risk of MM development (**Table 4**). We did not observe statistically significant differences between allele frequencies among MM patients and healthy blood donors (**Table 4**).

**Table 3:** Hardy−Weinberg equilibrium (HWE) for *ANGTPT2* variants in the case and control groups according to expected (E) and observed (O) values.

GROUPS	GENOTYPES of *ANGTPT2* variants			Total	HWE p value and χ^2^*
rs1868554					
**-**	**TT**	**TA**	**AA**	**-**	**-**
CONTROL					
E	54.67	52.65	12.67	120	p=0.66 χ^2^ = 0.19
O	56	50	14	120	
CASE					
E	54.61	40.77	7.61	103	p=0.02 χ^2^ = 4.73
O	49	52	2	103	
rs7825407					
	**GG**	**GC**	**CC**	–	–
CONTROL					
E	55.35	52.29	12.35	120	p=0.59 χ^2^ = 0.28
O	57	49	14	120	
CASE					
E	54.61	40.77	7.61	103	p=0.02 χ^2^ = 4.73
O	49	52	2	103	

**Table 4:** Comparison of the allele frequency and distribution of *ANGTPT2* variants among MM patients and controls.

Gene variants and alleles	MM n (%)	Controls n (%)	Odds ratio	95% CI	p values
rs1868554					
**DOMINANT**					
**TT**	49 (47.57)	56 (46.66)	1	–	–
**TA**	52 (50.48)	50 (41.66)	0.84	0.48-1.45	0.53
**AA**	2 (0.97)	14 (11.66)	6.12	1.32-29.28	0.02
**TA+AA**	54 (52.42)	64 (53.33)	1.03	0.61-1.75	0.88
**RECESSIVE**					
**TT+TA**	101 (98.05)	106 (88.32)	1	–	–
**AA**	2 (0.97)	14 (11.66)	6.66	1.47-30.08	0.01
**OVER DOMINANT**					
**TT+AA**	51 (48.54)	70 (58.32)	1	–	–
**TA**	52 (50.48)	50 (41.66)	0.70	0.41-1.19	0.18
**Total:**	103 (100%)	120 (100%)			
**T**	150 (73)	162 (67.5)	1	–	–
**A**	56 (27)	78 (32.5)	1.28	0.85-1.94	0.22
**Total:**	206 (100%)	240 (100%)			
rs7825407					
**DOMINANT**					
**GG**	49 (47.57)	57 (47.5)	1	–	–
**GC**	52 (50.48)	49 (40.83)	0.81	0.46-1.39	0.45
**CC**	2 (0.97)	14 (11.66)	6.01	1.30-27.79	0.02
**GC+CC**	54 (52.42)	63 (52.5)	1	0.59-1.69	1
**RECESSIVE**					
**GG+GC**	101 (98.05)	106 (88.33)	1	–	–
**CC**	2 (0.97)	14 (11.66)	6.67	1.47-30.08	0.01
**OVER DOMINANT**					
**GG+CC**	51 (48.54)	71 (59.16)	1	–	–
**GC**	52 (50.48)	49 (40.83)	0.67	0.39-1.15	0.14
**Total:**	103 (100%)	120 (100%)			
**G**	150 (73)	163 (68)	1	–	–
**C**	56 (27)	77 (32)	1.26	0.84-1.90	0.25
**Total:**	258 (100%)	240 (100%)			

### 3.2 Cytogenetic changes and ANGPT2 variants

The numbers of individuals with deletions of 17p13.1 and with chromosome 17 aneuploidies were low: n = 18 and n = 16, respectively. Deletion of 17p13.1 co-occurred with chromosome 17 aberrations. Among these changes, we observed chromosome 17 trisomy (n = 11), chromosome 17 tetrasomy (n = 1), chromosome 17 tetrasomy and deletion of two *TP53* alleles (n = 2), chromosome 17 trisomy and deletion of one *TP53* allele (n = 1), and chromosome 17 monosomy (n = 1). Due to the small number of particular chromosome 17 mutations, these changes were grouped and analyzed together. We did not observe an association between the studied variants and the presence of chromosome 17 aberrations—rs1868554, p=0.45 (OR=1.51, 95% CI 0.51-4.42); rs7825407, p=0.84 (OR=1.12, 95% CI 0.38-3.25). Due to the small number of individuals with structural cytogenetic changes, MM patients with del(17)(p13.1) containing the *TP53* gene *locus* and 14q32.2 translocations (with the *IgH* gene *locus*) were grouped and analyzed together. We did not observe an association between cytogenetic structural aberrations and the studied *ANGPT2* variants (p=0.33, OR=1.52, 95% CI 0.64-3.62).

### 3.3 ANGPT2 variants as risk factors for death and MM progression

Minor genotypes were analyzed together with heterozygotes due to their small sample size. Univariate Cox analysis revealed that patients with ISS stage III disease had a 2-fold (*p* = 0.001) increased risk of death (**Table 5**). In the case of MM patients who underwent autoHSCT, a lower risk of death was observed (HR = 0.238, *p* = 0.001). Similar findings were observed for disease relapse or progression in MM patients at stage III according to ISS (HR = 1.8, *p* = 0.001) and with autoHSCT (HR = 0.347, *p* = 0.001) (**Table 5**). Multivariate Cox regression analysis confirmed that patients who underwent autoHSCT had a decreased risk of death and disease relapse or progression (**Table 6**). In contrast, patients with stage III disease according to the ISS had an increased risk of death and disease relapse or progression. Univariate and multivariate Cox analyses did not reveal an impact of the studied genotypes on the risk of death, disease relapse or disease progression in MM patients.

**Table 5:** Univariate Cox analysis of the survival of MM patients.

Variable	Univariate Cox analysis for OS			Univariate Cox analysis for PFS		
	p value	HR	95% CI	p value	HR	95% CI
ISS						
I+II	–	R		–	R	–
III	0.001	2.138	1.347-3.395	0.001	1.808	1.290-2.533
AutoHSCT						
yes	0.001	0.238	0.101-0.564	0.001	0.347	0.187-0.643
no	–	R	–	–	R	–
rs1868554						
TT	–	R	–	–	R	–
TA+AA	0.825	1.077	0.558-2.080	0.377	0.789	0.466-1.335
rs7825407						
GG	–	R	–	–	R	–
GC+CC	0.792	1.093	0.566-2.111	0.402	0.799	0.472-1.351

**Table 6:** Cox multivariate analysis of the survival of MM patients.

Variable	Multivariate Cox analysis for OS			Multivariate Cox analysis for PFS		
	p value	HR	95% CI	p value	HR	95% CI
ISS						
I+II	–	R		–	R	–
III	0.030	1.7	1.052-2.747	0.038	1.464	1.022-2.098
AutoHSCT						
yes	0.019	0.33	0.131-0.831	0.017	0.438	0.223-0.860
no	–	R	–	–	R	–
rs1868554						
TT	–	R	–	–	R	–
TA+AA	0.969	1.07	0.031-36.70	0.884	0.788	0.032-19.510
rs7825407						
GG	–	R	–	–	R	–
GC+CC	0.949	0.89	0.026-30.26	0.979	0.958	0.039-23.533

### 3.4 Associations of the studied variants with clinical/laboratory values

We analyzed the potential relationships between clinical/laboratory results and selected genotypes of the *ANGPT2* gene. The comparative analysis did not reveal any statistically significant differences in laboratory or clinical parameters between patients with particular variants of polymorphisms rs1868554 and rs7825407 (**Table 7**).

**Table 7:** The clinical value of MM patients (at diagnosis) included in the study took into account the studied variants.

Variables	MM patients	rs1868554			rs7825407		
		TT	TA+AA	p value	GG	GC+CC	p value
**Mean age (years)**	65.02	63.84	66.11	0.23	63.71	66.22	0.20
**Free light chain ratio**	262.70	317.28	674.82	0.56	263.43	262.04	0.90
**% of plasma cells in bone marrow**	30.77	31.16	30.43	0.94	30.71	30.83	0.70
**Albumins (g/dL)**	3.63	3.58	3.66	0.77	3.55	3.69	0.67
**β2-microglobulin (mg/L)**	7.08	7.79	6.42	0.21	7.69	6.51	0.24
**Calcium (mM/L)**	2.45	2.52	2.42	0.17	2.51	2.43	0.26
**Hemoglobin (g/dL)**	10.46	10.20	10.68	0.12	10.15	10.73	0.08
**Creatinine (mg/dL)**	1.65	1.86	1.40	0.70	1.78	1.47	0.89
**Platelets (K/μL)**	210.11	208.96	211.17	0.99	205.31	214.48	0.62
**C-reactive protein (mg/L)**	12.65	13.43	11.76	0.40	14.10	11.15	0.23
**Estimated glomerular filtration rate mL/min/1.73m^2^**	68.95	71.21	66.99	0.37	73.31	65.08	0.13
**LDH (IU/L)**	347.24	347.96	346.61	0.58	341.25	352.57	0.26
*ANGPT2* expression*	0.73	0.67	0.80	0.89	0.62	0.84	0.61
**ANGPT2 level (in serum) pg/mL**	2994	2669	2907	0.52	2482	2995	0.32

*delta delta Ct.

### 3.5 ANGPT2 mRNA expression and clinical/laboratory values

The delta delta Ct method and log2-fold change methods were used to analyze *ANGPT2* expression. We observed a positive correlation between *ANGPT2* expression and CRP (C-reactive protein) levels (Spearman’s rho 0.26, p<0.05). A statistically significant negative correlation was found between *ANGPT2* expression and LDH levels (Spearman’s rho -0.25, p<0.05). In the case of other clinical/laboratory values, we did not observe statistically significant differences.

### 3.6 ANGPT2 serum concentration

The mean ANGPT2 concentration (pg/mL) in MM patients was greater than that in control individuals (2994 *vs*. 616, p < 0.001). Considering the analyzed variants, we did not observe an association between ANGPT2 concentration (pg/ml) and rs1868554 or rs7825407 (p = 0.52 or p = 0.32, respectively) (**Table 7**). Considering the rs1868554 and rs7825407 haplotypes, we observed a difference in ANGPT2 concentration at the level of tendency (p = 0.08) (**Table 8**). The analyzed haplotypes did not affect OS in MM patients (**Table 9**).

**Table 8:** Haplotypes comprising *ANGPT2* gene variants and their protein concentrations in serum (pg/mL).

rs1868554	rs7825407	Frequency	Mean concentration	Standard deviation	p value
TT	GG	0.46	2493	2492	0.08
TA	GC	0.50	3365	2443	

**Table 9:** Haplotypes comprising *ANGPT2* gene variants and OS.

rs1868554	rs7825407	Frequency	OS (months)	Standard deviation	p value
TT	GG	0.46	24.85	25.2	0.77
TA	GC	0.50	26.29	24.93	

### 3.7 Survival of MM patients considering the type of treatment and studied variants

**Figure 4:**
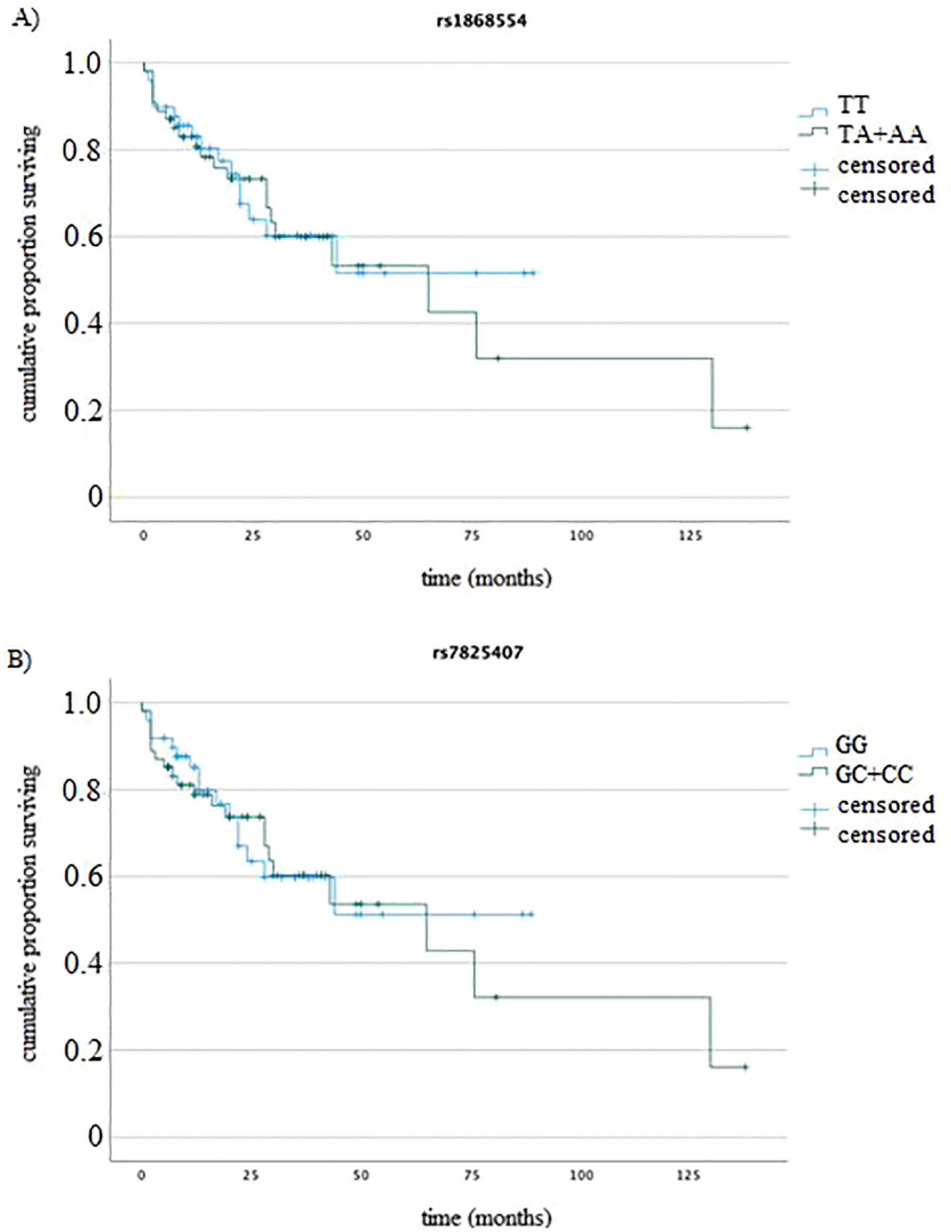
K−M analysis of OS **(A)** for rs1868554 (log-rank test *p* = 0.82) and **(B)** rs7825407 (log-rank test *p* = 0.79) according to genotype.

We analyzed the associations between the studied genotypes and the survival of MM patients (by log rank test). The rs1868554 and rs7825407 variants did not affect OS (log rank test p=0.82, p=0.79) or PFS (log rank test p=0.39 and p=0.36), respectively. **Figure 4** shows an example of the OS analysis including the *ANGPT2* variants (without considering the type of treatment).

## 4 Discussion

Angiogenesis is very important for the development and progression of malignant tumors. The formation of new blood vessels depends on the presence of proangiogenic factors. In MM, these compounds can be produced by both malignant cells and microenvironment bone marrow cells (35, 36).

In the course of multiple myeloma, the ANGPT2 factor plays an important role. Numerous studies indicate an increase in the concentration of ANGPT2 protein in the serum of MM patients compared to that in the serum of healthy donors (4, 9, 37). Our research aimed to first assess the serum ANGPT2 concentration in MM patients and healthy donors. Second, we investigated the effect of selected variants (rs1868554 and rs7825407) on *ANGPT2* expression at mRNA/the protein levels. Then, we checked whether the analyzed variants, gene expression and protein levels were related to the course of MM and the response to treatment.

In our study, we showed a significantly greater concentration of ANGPT2 protein in the serum of MM patients than in that of healthy blood donors. To date, several studies have been published with similar results (4, 9). However, we did not observe a correlation between the analyzed variants and *ANGPT2* gene expression at the mRNA or protein level. Considering the rs1868554 and rs7825407 haplotypes, we observed a difference in ANGPT2 concentration at the tendency level. The results of our study showed that individuals with the AA genotype of rs1868554 and the CC genotype of rs7825407 had a greater risk of developing MM. To date, no studies have been conducted to assess the contribution of these variants to MM progression. Two variants of the *ANGPT2* gene, rs1868554 and rs2442598, were significantly associated with acute lung injury at the II^nd^ clinical stage (20). Researchers are considering variants (other than those we analyzed) of the *ANGTPT2* gene that may have clinical relevance. Hu and colleagues examined five *ANGPT2* variants, rs2442598, rs734701, rs1823375, rs11137037, and rs12674822, and their impact on the development and course of lung cancer (38). They showed that carriers of the GT allele (rs12674822) had a greater risk of lung cancer than did carriers of the wild type, GG. The presence of the CC genotype in rs11137037 was associated with a greater degree of clinical disease advancement in comparison to carriers of the AA genotype (38). Hu and colleagues examined the effect of *ANGPT2* variants on susceptibility to developing breast cancer in a Chinese Han population. Five *ANGPT2* variants were selected: rs2442598, rs734701, rs1823375, rs11137037 and rs12674822. Based on the research results, the authors concluded that the T-T-C-A-T *ANGPT2* haplotype significantly increased the risk of breast cancer development by almost 1.39 times (39). Our next study can be expanded to include *ANGPT2* variants, which we have not yet analyzed. It is possible that they are related to elevated angiopoietin-2 levels in MM patients.

In the present study, genetic changes did not affect the expression of the *ANGPT2* gene at the mRNA or protein level. However, *ANGPT2* expression (mRNA level) was positively correlated with CRP and negatively correlated with LDH in our MM patients. Several acute phase proteins, e.g., CRP or IL-6, predict MM prognosis and may influence the survival of MM patients (40, 41). Additionally, it has been shown that a high CRP level at the time of MM diagnosis is a factor increasing the risk of cachexia during treatment (42).

According to the observations of Pappa et al., ANGPT2 seems to play a crucial role in the angiogenic process in MM patients, with a very important impact on their prognosis (4). They showed that the ANGPT2 concentration and BM MVD were greater in MM patients than in controls (4). In our study, we did not examine the MVD or ANGPT2 levels in the bone marrow plasma. MVD is not routinely performed in patients with MM, and it is currently impossible to perform this test because most of the examined patients died. We collected bone marrow aspirates and sera from the time of disease progression to determine changes in ANGPT2 expression (at the mRNA and protein levels). Saltarella et al. reported no significant differences between the blood and bone marrow plasma levels of proangiogenic cytokines, e.g., ANGPT2, HGF, VEGF or TNF-α, in MM patients (43). This is inconsistent with our results of ANGPT2 serum levels in MM patients. Joshi et al. reported significant increases in ANGPT2 and VEGF concentrations and their correlation with disease severity in patients with MM (9).

The protein ANGPT2 is a proangiogenic factor. This means that it supports the development of new blood vessels, or the process of neoangiogenesis. The formation of blood vessels in niches where MM cells are located is a necessary process for their proliferation and spread. Higher levels of ANGPT2 protein in people with multiple myeloma have a negative effect on the general condition of the patient because it promotes disease progression. Survival analysis of MM patients, conducted by Pappa et al. showed that patients with higher serum ANGPT2 levels before treatment were accompanied by shorter survival compared to those with lower values (62 *vs*. 36 months) (p < 0,04) (4).

One of the more important findings described in the literature is that *ANGPT2* gene expression is largely dependent on the DNA methylation state *ANGPT2* gene expression could be directly regulated by DNA methylation. The increase in the methytulation of the regulatory part of the gene can cause inhibition of its expression. Methylation levels of promoter region in *ANGPT2* gene have been reported to be negatively correlated with its mRNA expression in CLL patients. This finding suggesting that *ANGPT2* promoter methylation leads to gene silencing (44, 45). The group of epigenetic factors that affect gene expression also includes microRNA. miR-135b, miR-16 and miR-15a are involved in the regulation of the expression of genes involved in angiogenesis (46, 47). miRNA molecules attach to mRNA molecule and, as a consequence, completely or partially block protein translation. miR-16 and miR-15a are involved in the regulation of VEGF-A expression and miR-135b are involved in the regulation of the HIF-1 factor (46, 47). miRNA molecules that can regulate *ANGPT2* gene expression include miR-542-3p (48). He and coworkers described, that miR-542-3p inhibit translation of Angpt2 mRNA by binding to its 3-UTR, and addition of miR-542-3p to *in vitro* cultured endothelial cells attenuate angiogenesis. Another miRNA molecule that can inhibit *ANGPT2* gene expression is miR−135a−5p (49). Using a luciferase activity assay, Diao et coworkers identified that *ANGPT2* is a potential target gene of miR−135a−5p in gallbladder cancer cells (49).

Furthermore, the expression of the *ANGPT2* gene in MM may be affected by many factors: signals from plasma cells, bone marrow microenvironmental cells, *ANGPT2* gene variants and epigenetic modifications (11, 20, 44). This system is very complex, and much research is still needed to elucidate the biology of MM cells.

The group of patients included in our study were diagnosed between 2013 and 2020, when standard treatment included the use of thalidomide and bortezomib. The antiangiogenic and immunomodulatory effects of thalidomide resulted in the inclusion of this drug in the therapy of MM patients several years ago. Thalidomide belongs to a group of immunomodulatory drugs (IMiDs). IMiDs play a crucial role in the treatment landscape across various stages of MM. The mechanism of action of IMiDs is still unclear. Thalidomide and its analogues affect angiogenesis indirectly. Ito and coworkers identified cereblon as a teratogenic target of thalidomide (50). Cereblon is the substrate receptor of the E3 ubiquitin ligase. Inhibition of cereblon expression in human multiple myeloma cell lines significantly reduces cell growth and viability. This data suggesting a key role of E3 ubiquitin ligase in mediating the IMiDs activity (50, 51). Research data on IMiDs and bone marrow angiogenesis in patients with MM are contradictory. Data from the publications Kumar et al., and Hatjiharissi et al., showed that MVD in bone marrow is significantly reduced in patients treated with thalidomide alone or in combination with dexamethasone, without a decrease serum levels of angiogenic cytokines e.g. VEGF, b-FGF, IL-6 (52, 53). Contrary results were presented in publication by Cury and colleagues. These scientists demonstrated the absence of any decrease in bone marrow angiogenesis in patients treated with thalidomide (54). We were unable to find a publication in which the direct effect of thalidomide on the expression of the *ANGPT2* gene was described. This gives us the opportunity to focus our research for the future. Especially considering functional studies *in vitro*.

In an *in vitro* study, Saha and colleagues reported that thalidomide has antiangiogenic effects by targeting VEGFR-2 Tyr-1175, a key autophosphorylation site that regulates proangiogenic responses (55). However, Peach and colleagues previously showed that the molecular target of IMiDs (including thalidomide) is the cereblon protein (56). Wang and colleagues used a model based on human umbilical vein endothelial cells (HUVECs) in their angiogenesis study (57). Researchers have shown that thalidomide can inactivate the endothelium by downregulating angiogenic factors. Thalidomide significantly decreased ANGPT2 and VEGF protein expression and reduced *ANGPT2* expression at the mRNA level. However, ANGPT1 concentrations did not significantly change (50). In our study, we did not observe an association between *ANGPT2* variants and OS or PFS considering treatment. Moreover, *ANGPT2* haplotypes were not related to the OS of MM patients.

In summary, angiogenesis is an essential process for the development of multiple myeloma. Proangiogenic factors may predict the course of the disease toward progression. Detailed studies of the factors involved in this process are necessary to expand our knowledge of the biology of MM.

Aberrant angiogenesis in bone marrow is a key hallmark of multiple myeloma progression and this process is very complex. Both MM and BMSCs cells play essential role to shape the BM angiogenic niche. This leading to the formation of new blood vessels by recruiting endothelial progenitor cells (EPCs) (58). During the activation of angiogenesis, high expression of oncogenes such as c-myc and Jun-B induces plasma cells to secrete high amounts of pro-angiogenic cytokines, including: VEGF, FGF-2, HGF, ANGPT2 and IGF-1 (59). In response to released factors, ECs increase the expression of receptors including: VEGFR, HGFR, and FGFR2 (58). The migration of other immune system cells also increases (58). Already in 2013, Bhaskar and colleagues, based on their research, suggested the important role of angiopoietin in the etiology and course of MM and suggested ANGPT2 as a potential target for antiangiogenic therapy in the treatment of MM (60). ANGPT2 is the vascular growth factor, mediated through VEGF-independent pathways (61). However, in subsequent studies, the researchers showed that VEGF and ANGPT2 play synergistically, and their expression levels are predictive of PFS in MM patients (60). Rao and colleagues described the involvement of the HB-EGF-EGFR pathway in MM-associated angiogenesis (62). They demonstrated that HB-EGF and EGFR are highly expressed in bone marrow endothelial cells of MM patients and are potent inducers of angiogenesis (62). In addition, they showed that blockade of the HB-EGF–EGFR pathway using EGFR inhibitors (such as erlotinib) reduced angiogenesis and tumor growth in *in vitro* and *in vivo* models of MM. These results suggest that targeting multiple angiogenic pathways, including ANGPT2, VEGF, and HB-EGF–EGFR signaling, may provide a more comprehensive therapeutic strategy for MM. Vanucizumab (RO5520985) is a novel bispecific humanized immunoglobulin that acts as a dual targeting inhibitor of VEGF-A and ANGPT2 (63, 64). Clinical studies have been conducted on the efficacy of vanucizumab in solid tumors (64, 65). There are no clinical studies with vanucizumab in hematological malignancies.

In the expression of proangiogenic factors are involved microenvironment cells, genetic variants and epigenetic factors such as miRNA molecules. This process is very complex and it is incredibly difficult to find one pathway that plays a major role in the pathogenesis of BM angiogenesis in multiple myeloma. This shows the need to construct complex therapies that will target different points of the disease, which are the proliferation of plasmocyte cells and angiogenesis of the microenvironment of bone marrow niches. Analysis of 3 markers of angiogenesis: ANGPT2, VEGF and HB-EGF can become a prognostic panel to assess the course of MM and predict the use of angiogenesis-oriented therapy.

The limitation of our study is the relatively small sample size, which is partly due to the low incidence of MM. Further analysis with a larger cohort may help to better understand the significance of *ANGPT2* variants in MM pathology. Another limitation is the small number of ANGPT2 variants that we selected for this study. This choice was made taking into account their sequence location. Studies with selected variants have not been conducted in MM thus far. In the present study, we did not include analyses of MVD, VEGF variants or expression (at the mRNA and protein levels), or other *ANGPT2* variants with confirmed significance in hematologic malignancies. Moreover, we did not perform an analysis of *ANGPT2* expression during MM progression or after treatment. Such an analysis would allow us to obtain much more data and could show how *ANGPT2* gene expression changes depending on the applied therapy. Our study did not include a group of patients with relapsed or refractory MM. Including this group, could provide insight into how *ANGPT2* variants affect disease behavior and treatment response at different stages of the disease. In the future, we plan to expand the study group to at least 250 patients. A good direction is to cooperate with centers from another part of Europe to obtain samples. This will create a more diverse group. We plan to study other variants of the *ANGPT2* gene, include variants and expression of the VEGF gene. In addition, we would like to complete the research with a functional study on cell lines, for example to study the effect of angiogenesis inhibitors on MM cell survival, including vanucizumab.

## 5 Conclusion

Taken together, the results of our study suggest that *ANGPT2* variants may have negative prognostic implications for the risk of developing MM. Moreover, our results showed that *ANGPT2* expression at the mRNA level correlates with CRP, a negative prognostic factor in MM. The ANGPT2 protein is a proangiogenic factor, and its concentration is significantly greater in MM patients than in healthy individuals, which was also confirmed in our research. Therefore, this protein with VEGF and HB-EGF, should be considered in the future as a markers of angiogenesis in MM.

## Data Availability

The raw data supporting the conclusions of this article will be made available by the authors, without undue reservation.
